# The (Glg)ABCs of cyanobacteria: modelling of glycogen synthesis and functional divergence of glycogen synthases in *Synechocystis* sp. PCC 6803

**DOI:** 10.1002/1873-3468.70299

**Published:** 2026-02-03

**Authors:** Kenric Lee, Dimitrios Bekiari, Sofia Doello, Karl Forchhammer

**Affiliations:** ^1^ Interfaculty Institute of Microbiology and Infection Medicine University of Tübingen Germany

**Keywords:** biochemical assay, cyanobacteria, enzyme coupling, flux control, GlgA regulation, glycogen synthesis model, metabolic engineering, stoichiometry

## Abstract

Glycogen is the principal carbon reserve in *Synechocystis* sp. PCC 6803. We reconstituted its biosynthetic pathway *in vitro*—GlgC (glucose‐1‐phosphate adenylyltransferase), two glycogen synthase isoenzymes (GlgA1, GlgA2) and the branching enzyme GlgB—to define how supply, polymerization and branching set flux and product structure. GlgA2 shows higher specific activity and cooperates with GlgB‐generated branched primers, whereas GlgA1 has higher substrate affinity and responds more to primer concentration. Product profiling links mechanism to architecture: GlgA1 produces more‐branched glycogen, while GlgA2 yields longer, less‐branched polymers, with GlgB biasing utilization toward GlgA2. The complementary behaviors of GlgA1 and GlgA2 provide capacity for rapid accumulation versus steady‐state maintenance and offer dynamic metabolic levers to tune glycogen content and architecture in cyanobacteria.

## Graphical abstract

We reconstituted *Synechocystis* glycogen synthesis *in vitro* from purified enzymes and showed that two GlgA isoenzymes produce glycogen with different architectures: GlgA1 yields denser, highly branched glycogen, whereas GlgA2 synthesizes longer, less‐branched chains. Glycogen synthesis requires a primer, and GlgB is essential—without GlgB, both GlgA make amylose‐like linear polymers. The overall glycogen synthesis pathway flux is GlgA‐limited.

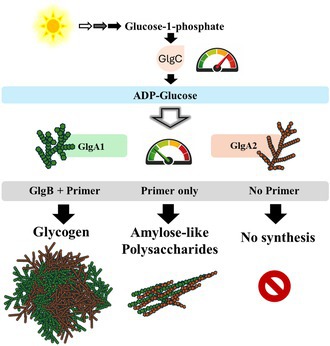

## Abbreviations

**3‐PGA**, 3‐phosphoglycerate

**ADP‐Glc**, ADP‐Glucose

**Cα RMSD**, Cα root‐mean‐square deviation

**Glc‐1P**, Glucose‐1‐phosphate

**Glc‐6P**, Glucose‐6‐phosphate

**GlgA**, Glycogen Synthase

**GlgB**, Glycogen branching enzyme

**GlgC**, Glucose‐1‐phosphate adenylyltransferase

**LDH**, Lactate dehydrogenase

**MG**, Malachite Green (Assay)

**PEP**, Phosphoenolpyruvate

**PGM**, Phosphoglucomutase

**Pi**, Inorganic orthophosphate

**PK**, Pyruvate kinase

**PPi**, Pyrophosphate

***Synechocystis***, *Synechocystis* sp. PCC 6803

Cyanobacteria are a large group of photoautotrophic prokaryotes, of which some species are also capable of heterotrophic growth. Owing to their adaptability, representatives of cyanobacteria have been found to be successful in nearly every biome on the planet and are regarded as the precursors to higher plants [1]. Glycogen is the principal carbon storage polysaccharide in cyanobacteria, playing a central role in cellular energy homeostasis and adaptation to fluctuating environmental conditions [2, 3, 4].

In *Synechocystis* sp. PCC 6803 (hereafter *Synechocystis*), the pathway for glycogen biosynthesis is orchestrated by a suite of specialized enzymes [5]. Glycogen synthesis begins with the starting molecule, glucose‐1‐phosphate (Glc‐1P) which is interchangeably converted from glucose‐6‐phosphate (Glc‐6P) mainly by phosphoglucomutase (PGM, product of *sll0726*, EC:5.4.2.2). Glc‐1P is then utilized in the initial, rate‐limiting step catalyzed by glucose‐1‐phosphate adenylyltransferase (hereafter GlgC, product of *slr1176*, EC:2.7.7.27), which synthesizes ADP‐glucose (ADP‐Glc) from ATP and Glc‐1P—a process subject to intricate allosteric regulation by metabolic effectors such as 3‐phosphoglycerate (3‐PGA) and inorganic phosphate (Pi) [6, 7, 8, 9]. The ADP‐Glc generated then serves as the activated glucosyl donor for glycogen synthase (hereafter GlgA) enzymes (EC:2.4.1.21), which directly catalyze the elongation of a growing glycogen chain. These processes mirror the complexity observed in higher plants and shed light on evolutionary parallels in α‐glucan metabolism [5].

*Synechocystis* encodes two distinct GlgA isoenzymes, GlgA1/*sll0945* and GlgA2/*sll1393*. Unlike most bacteria, which typically possess a single GlgA, these isoforms display complementary and, in some contexts, specialized roles in glycogen polymerization and chain elongation [5]. Recent studies indicate that GlgA1 and GlgA2 differ in their chain extension properties—GlgA1 being distributive and GlgA2 more progressive—affecting the fine structure and branching patterns of the resulting glycogen granules [5, 10]. These differences suggest isoenzyme divergence for nuanced physiological or metabolic adaptation and underscore the need for a systematic characterization of their respective biochemical properties. The final step in the maturation of glycogen involves the glycogen branching enzyme (product of *sll0158*, EC:2.4.1.18, hereafter GlgB), which inserts α‐1,6‐glycosidic linkages, increasing the number of non‐reducing ends and enhancing the water solubility of the polymer [11, 12]. The interplay between GlgA and GlgB is a key determinant of the overall structure, size, and accessibility of glycogen particles, which in turn can influence the cell's capacity for rapid carbon mobilization under stress or during recovery from nutrient deprivation [13, 14].

Despite these advances, understanding of how GlgA isoenzymes coordinate with GlgC and GlgB remains incomplete. Questions persist regarding their kinetic properties, primer utilization preferences, differential regulation by allosteric metabolites, and the structural outcomes of their activity under varying enzymatic and substrate conditions. Dissecting these parameters is critical for comprehending not only glycogen metabolism in cyanobacteria but also its parallels with starch biosynthesis in plants and the broader evolutionary context of polysaccharide storage in photoautotrophic organisms.

In this work, we establish an integrated *in vitro* model for *Synechocystis* glycogen synthesis, leveraging precise biochemical assays to dissect the individual and collective roles of GlgA1, GlgA2, and GlgB. Our work aims to clarify substrate selectivity, primer dependency, regulatory mechanisms, and product structure, providing new insights into the functional divergence of GlgA isoenzymes and their physiological significance.

## Methods

### Culture and growth conditions of *E. coli*

Unless otherwise specified, the cultivation of all strains was performed as previously reported [6]. All strains used in this study are listed in Table 1.

**Table 1:** *E. coli* and *Synechocystis sp*. PCC 6803 strains used in this work.

Strain	Genotype/references
*E. coli* NEB10β	Δ(ara‐leu) 7697 araD139 fhuA ΔlacX74 galK16 galE15 e14‐ φ80dlacZΔM15 recA1 relA1 endA1 nupG rpsL (StrR) rph spoT1 Δ(mrr‐hsdRMS‐mcrBC)
*E. coli* Rosetta‐gami (DE3)	Δ(ara‐leu)7697 ΔlacX74 ΔphoA PvuII phoR araD139 ahpC galE galK rpsL (DE3) F′[lac+ lacIq pro] gor522::Tn10 trxB pRARE2 (CamR, StrR, TetR)
*Synechocystis* sp. 6803 WT‐GT	From Chen *et al*. [49], Glucose tolerant wild‐type strain
*Synechocystis* sp. 6803 Δ*glgA1*	From Gründel *et al*. [50], WT‐GT Background, *sll0945::km* ^*R*^
*Synechocystis* sp. 6803 Δ*glgA2*	From Gründel *et al*. [50], WT‐GT Background, *sll1393::cm* ^*R*^

### Culture and growth conditions of *Synechocystis* sp. PCC 6803

The cultivation of all cyanobacterial strains was performed as previously described [14]. All strains used in this study are listed in Table 1.

### Expression and purification of recombinant his‐tagged glycogen synthesis enzymes

The assembly of the expression constructs of N‐terminal His‐tagged GlgA1 and GlgC proteins was performed previously as described [6].

The GlgA2 gene was amplified from *Synechocystis* sp. PCC 6803 genomic DNA using the following primers; forward: 5′‐GCCTGGTGCCGCGCGGCAGCATGTACATCGTTCAAATTGCCTCAGAATGCGC‐3′ and reverse: 5′‐TGTCGACGGAGCTCGAATTCTTAAGCCCGGATGTATTCGTAGGCTTCCACA‐3′. The expression plasmid pET28a was similarly linearized and amplified using the following primer pair; forward: 5′‐GCAATTTGAACGATGTACATGCTGCCGCGCGGCACC‐3′ and reverse: 5′‐ACGAATACATCCGGGCTTAAGAATTCGAGCTCCGTCGACAAGCTTGCGGC‐3′.

Similarly, the GlgA gene from *E. coli* (GlgA^*E.coli*^) was amplified from genomic DNA using the following primers; forward: 5′‐GCCTGGTGCCGCGCGGCAGCATGCAGGTTTTACATGTATGTTCAGAGATGTTCCCG‐3′ and reverse: 5′‐TGTCGACGGAGCTCGAATTCCTATTTCAAGCGATAGTAAAGCTCACGGTACGACTTCG‐3′. The expression plasmid pET28a was similarly linearized and amplified using the following primer pair; forward: 5′‐TTTACTATCGCTTGAAATAGGAATTCGAGCTCCGTCGACAAGCTTGC‐3′ and reverse: 5′‐CATACATGTAAAACCTGCATGCTGCCGCGCGGCACC‐3′.

The subsequent assembly of the pET28a expression constructs for GlgA2 and GlgA^*E.coli*^ was carried out in the same manner as for GlgA1 and GlgC. The subsequent induction and expression of recombinant proteins were performed using IMAC as previously described [6]. Cell lysis was carried out in lysis buffer (20 mm Tris/HCl pH 7.8, 50 mm NaCl, 5 mm MgCl_2_, 40 mm imidazole) supplemented with CellLytic buffer, Benzonase® (both from Merck, Darmstadt, Germany), lysozyme and one tablet of cOmplete™ protein inhibitor cocktail (Roche, Mannheim, Germany). The lysate was clarified via ultracentrifugation at 16 000 ***g***, 30 min at 4 °C and filtered using a 0.22 μm syringe filter prior to column application.

The recombinant proteins were purified via immobilized metal affinity chromatography (IMAC) followed by a polishing step using anion exchange to yield proteins of a high purity. All purifications steps were performed using the ÄKTA purifier chromatography system (Cytiva, Freiburg, Germany). IMAC was performed with Ni‐NTA HisTrap columns (Cytiva) according to the manufacturer's directions. The His‐Tagged proteins were eluted with a 30 CV gradient of 40 mm to 500 mm imidazole in a buffer containing 20 mm Tris/HCl pH 7.8, 500 mm NaCl. Peak fractions were identified based on their UV 280 nm signal and pooled.

Buffer exchange of the pooled fractions was performed with the Amicon® Ultra Centrifugal Filter, 30 kDa MWCO (Merck) with two washes with IEX buffer (20 mm Tris/HCl pH 7.8) prior to anion exchange with HiTrap Q High Performance columns (Cytiva) according to the manufacturer's directions. The proteins were eluted with a 30 CV gradient of 0–750 mm NaCl in IEX buffer (20 mm Tris/HCl pH 7.8). As before, fractions from the largest peak were collected and buffer exchange was performed with two washes of concentration buffer (20 mm Tris/HCl pH 7.8, 150 mm KCl, 1 mm EDTA) followed by exchange into storage buffer (20 mm Tris/HCl pH 7.8, 150 mm KCl, 1 mm EDTA, 50%_(v/v)_ glycerol).

### Expression and purification of recombinant strep‐tagged proteins

The construct for recombinant C‐terminal Strep II‐tagged GlgB was purified as previously described [15]. PGM was purified as previously described [16].

### ADP/NADH coupled assay for GS activity (Assay A)

Glycogen synthase activity was assayed as previously described using a modified spectrophotometric method from that presented by Wayllace *et al*. [6, 17]. The following assay parameters were changed to suit the requirements for this study. The components of the assay in a total volume of 100 μL per reaction were; assay buffer (50 mm HEPES‐NaOH, pH 8.0, 12 mm MgCl_2_) with 10 U·mL^−1^ Lactate Dehydrogenase (LDH), 9 U·mL^−1^ Pyruvate Kinase (PK), 1 mm Phosphoenolpyruvate (PEP), 0.4 mm NADH, 1 mg mL^−1^ Bovine Glycogen and 200 nm GlgA1 or GlgA2. The reaction was assayed in 96‐well clear bottom plates at 30 °C for variable durations, depending on the experimental design. Absorbance was measured using Tecan Spark 10 m (Tecan, Männedorf, Switzerland) with readings taken every minute at 340 nm. Each standard reaction was started with the addition of the substrate ADP‐Glc.

### Glycogen synthesis and precipitation (assay B)

The synthesis of glycogen was performed in a similar manner to that of assay A with several modifications. The components of the assay in a total volume of 5 mL per reaction were as follows: assay buffer (50 mm HEPES‐NaOH, pH 8.0, 12 mm MgCl_2_) with 4 mm ATP, 4 mm 3‐PGA, 4 mm Glc‐1P and 200 μg·mL^−1^ starch as a primer. About 9 U·mL^−1^ PK and 2 mm PEP were also added to maintain ATP concentrations for optimal GlgC activity. Glycogen synthesis enzymes were added in a concentration ratio of 200 nm monomeric GS to 200 nm tetrameric GlgC, with and without 20 nm of GlgB. The reaction was left to run overnight (~20 h) at 30 °C with gentle agitation on a shaker.

The next day, all samples were heated at 90 °C for 10 min to inactivate the protein components of the reaction, followed by centrifugation at 17 000 ***g***, 4 °C for 30 min to separate the insoluble fraction of the reaction. The supernatant was carefully decanted and mixed with 4 volumes of ice‐cold absolute ethanol before being stored overnight at −20 °C to precipitate the reaction products. The precipitate was then pelleted via centrifugation at 17 000 ***g***, 4 °C for 15 min and washed twice with 70% ethanol and 100% ethanol before being left to dry completely in a 60 °C oven. The dried products were resuspended in distilled water, and an additional filtration step was performed using Amicon® Ultra Centrifugal Filter, 3 kDa MWCO (Merck) with four washes of distilled water to remove soluble contaminants. The remaining concentrate was resuspended in distilled water for further analysis.

### PGM forward reaction assay (Assay C)

The PGM forward reaction assay was adapted from Assay A and the coupled assay described in our previous report [6]. Each reaction (100 μL total volume) contained assay buffer (50 mm HEPES‐NaOH, pH 8.0, 12 mm MgCl_2_), 40 μm Glc‐1,6‐phosphate, 2 mm ATP, 2 mm 3‐PGA, 1 mm PEP, 0.4 mm NADH, and 1 mg·mL^−1^ bovine glycogen as primer, together with the coupling enzymes LDH and PK as specified above. Glycogen synthesis was catalyzed by 400 nm monomeric GlgA^*E.coli*^ and 400 nm tetrameric GlgC in the presence of PGM. Reactions were performed in 96‐well clear bottom plates as described previously and initiated by the addition of Glc‐6P.

### GlgC tetramer concentration ratios required to match GlgA activity under assay conditions

We calculated the theoretical GlgA–GlgC stoichiometries on a flux basis using the intrinsic specific activities from Assay A (GlgA1, GlgA2, GlgA^*E. coli*^ with ADP‐Glc) and the previously reported intrinsic GlgC activity from the MG assay [6] (Table 2). For a given enzyme, the flux was expressed in μm min^−1^ per nanomolar enzyme, as $J=V_{\max}\cdot \mathrm{MW}\cdot 10^{-6}$, where *V*_max_ was expressed in U·mg^−1^, MW is the molecular weight of GlgA monomers or GlgC tetramers in g mol^−1^, and 1 U = 1 μmol·min^−1^. For GlgC, the supply capacity was computed as *J*_*C*_, while for each GlgA isoform the consumption capacity per nm was computed as *J*_A_. The GlgA: GlgC stoichiometric ratio required to match the GlgA demand was then obtained as $A:C_{\text{ideal ratio}}=\frac{J_{C}}{J_{A}}$ and the corresponding ideal concentration of GlgC required was calculated as ${\left[C\right]}_{V_{\max_{A}}}=\frac{\left[A\right]}{A:C_{\text{ideal ratio}}}$. These calculations were used to compare the intrinsic supply capacity of GlgC with the utilization capacities of GlgA1, GlgA2, and GlgA^*E. coli*^, and to interpret the minimum GlgC concentration required for a stable supply of ADP‐Glc to GlgA under assay conditions. Table 2 summarizes all relevant parameters for this calculation.

**Table 2:** Experimental assay parameters and enzyme concentrations with molecular weights.

Enzyme	Molecular weight [g mol^−1^]	Substrate	Spec. activity [U·mg^−1^]	Flux capacity [μm·min^−1^ per nm]	*K*_m_ [μm]	Optimal GlgA: GlgC ratio
GlgC Tetramer	200 856.64	Glc‐1P	8.90	1.79	240 ± 50	—
GlgA1	56 372.5	ADP‐Glc	0.51	2.86 × 10^−2^	152 ± 3.6	62 : 1
GlgA2	58 251.0	ADP‐Glc	0.77	4.49 × 10^−2^	219 ± 9.9	40 : 1
GlgA^*E.coli*^	54 848.6	ADP‐Glc	538	2.95 × 10^1^	275 ± 17	0.06 : 1

### Glycogen purification from *Synechocystis* cultures

Exponential *Synechocystis* cultures of 300 mL at ~0.8 OD_750_ were harvested via centrifugation and washed twice with 30%_(w/v)_ KOH. The washed cell pellets were resuspended in 30%_(w/v)_ KOH and heated at 85 °C for 30 min for cell lysis. After lysis, the precipitation of glycogen was carried out as previously described; the lysate was centrifuged at 17 000 ***g***, 4 °C for 30 min to separate cell debris. The supernatant was carefully decanted and mixed with 4 volumes of ice‐cold absolute ethanol before being stored overnight at −20 °C to precipitate glycogen. The precipitate was then pelleted via centrifugation at 17 000 ***g***, 4 °C for 15 min and washed twice with 70% ethanol and 100% ethanol before being left to dry completely in a 60 °C oven. The dried products were resuspended in distilled water and an additional filtration step was performed using Amicon® Ultra Centrifugal Filter, 3 kDa MWCO (Merck) with four washes of distilled water to remove soluble contaminants. The remaining concentrate was resuspended in distilled water for further analysis.

### GS reaction product quantification and analysis (spectrophotometric quantification: Iodine‐iodide assay)

The quantification of the *in vitro* reaction products was performed by mixing the reaction products with 70 μL of iodine solution (2 mm I_2_, 30 mm KI) in a total volume of 150 μL. The reaction products were compared to glycogen or starch standard solutions in the concentration range of 0–2 mg·mL^−1^ based on their absorbances at wavelengths of 490 nm or 560 nm for glycogen and starch, respectively. For the measurement of the reaction product absorbance spectra, the concentration of the reaction products was adjusted to 0.5 mg·mL^−1^, and the absorbance was measured from 400 nm to 700 nm. Measurements were performed using the Tecan Spark 10 m (Tecan).

### Fast glycogen measurement assay (enzymatic quantification)

The enzymatic determination of glycogen was performed with the method presented by Vidal et al. with some minor modifications and was used to validate our Iodine‐Iodide assay described above [18]. The total assay volume was modified to 100 μL, consisting of 30 μL enzymatic mix and 70 μL of glycogen sample from Assay B. The concentration of the glycogen sample was previously determined using the Iodine‐Iodide assay and was used for comparisons. All measurements were done using the Tecan Spark 10 m (Tecan).

### Figures, bioinformatics, and analyses

All data were analyzed and visualized using GraphPad Prism 10 (GraphPad, Boston, MA, USA). Primer and expression construct designs were carried out *in silico* using SnapGene (GSL Biotech, San Diego, CA, USA). Multiple sequence alignment was generated in ClustalW using the UniProt GlgA sequences, and structure‐guided motif mapping was performed in UCSF ChimeraX by superposing AlphaFold3 GlgA models with Matchmaker and extracting alignment‐equivalent residues from the resulting structure‐informed sequence alignment. Cα RMSD (Cα–root‐mean‐square deviation) analysis of superimposed models was also performed in UCSF ChimeraX [19, 20, 21].

## Results

### Comparable catalytic efficiencies of GlgA1 and GlgA2

**Fig. 1:**
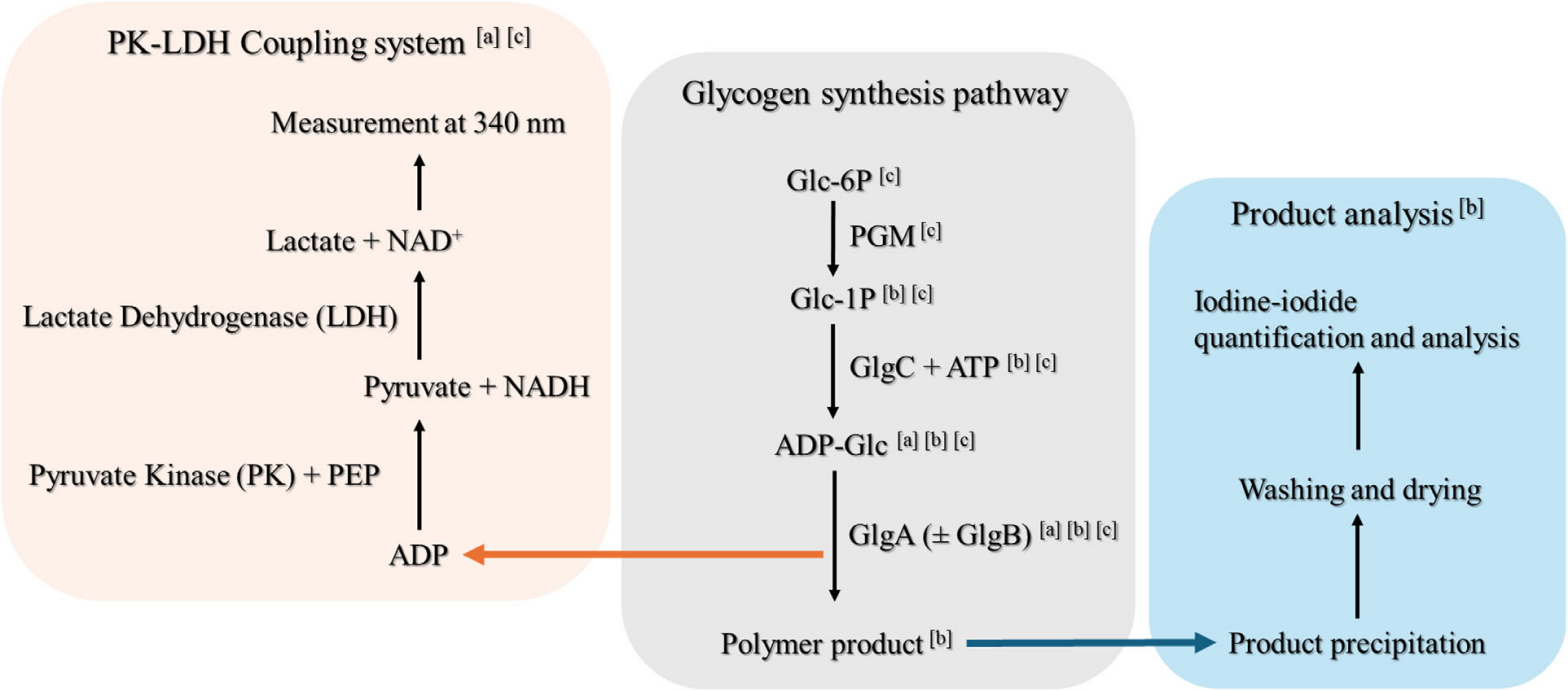
Schematic overview of the glycogen synthesis pathway in *Synechocystis*, and methods used in this study to assay different components of the pathway. Indicated are components [a] involved in Assay A [b] involved in Assay B [c] involved in Assay C. The details of each assay are given in their respective sections in the methods.

**Fig. 2:**
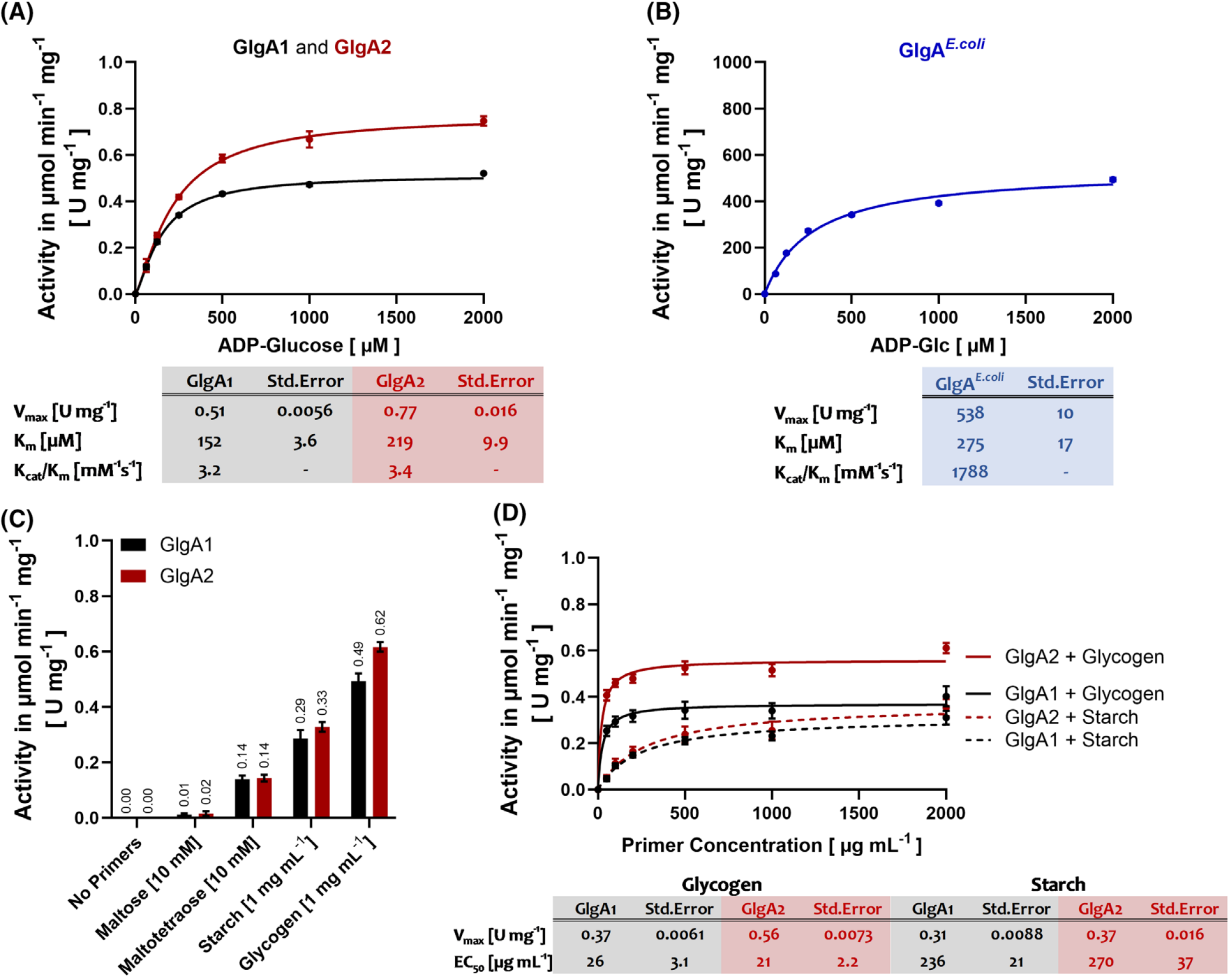
GlgA Kinetics assayed using the ADP/NADH Coupled Assay for glycogen synthesis Activity (Assay A). (A) ADP‐Glc titration with 200 nm GlgA1 or GlgA2. About 1 mg·mL^−1^ glycogen was used as a primer. (B) ADP‐Glc titration with 1 nm GlgA^*E.coli*^. 1 mg·mL^−1^ glycogen was used as a primer. (C) Comparison between synthesis primers. The reaction was started with 1 mm ADP‐Glc. (D) Titration curves for glycogen and starch. As above, the reaction was started with the addition of 1 mm ADP‐Glc. Where applicable, each data point represents the mean of replicates with the SD shown as error bars (*n* = 3–12).

Recombinant His‐tagged GlgA1, GlgA2 and GlgC, and Strep‐tagged GlgB were expressed in *E. coli* and purified to near‐homogeneity by affinity chromatography followed by anion exchange. Purified proteins were recovered at the expected molecular weights and were used for kinetic assays. We first revisited the kinetic properties of the GlgA isoenzymes by initiating glycogen synthesis with direct addition of ADP‐Glc and using bovine glycogen as the primer (Assay A) GlgA activity was monitored via a well‐documented coupled spectrophotometric assay detecting ADP release through pyruvate kinase (PK) and lactate dehydrogenase (LDH) activities in the presence of phosphoenolpyruvate (PEP) and NADH (Fig. 1) [6, 17]. As shown in Fig. 2A, GlgA2 (0.77 ± 0.02 U·mg^−1^) exhibited approximately 50% higher activity than GlgA1 (0.51 ± 0.01 U·mg^−1^), albeit with a slightly higher *K*_m_ for ADP‐Glc (GlgA2 *K*_m_: 219 ± 9.9 μm; GlgA1 *K*_m_: 152 ± 3.6 μm). The resulting catalytic efficiency (*K*_cat_/*K*_m_) of GlgA1 was calculated to be 3.2 mm ^−1^ s^−1^ which is comparable to GlgA2 at 3.4 mm ^−1^ s^−1^. To further validate our assay method, we performed parallel ADP‐Glc titration replacing the *Synechocystis* GlgA isoenzymes with *E. coli* GlgA (GlgA^*E.coli*^) under identical reaction conditions (Fig. 2B). Notably, GlgA^*E.coli*^ exhibited enzymatic activity approximately 10^3^‐fold higher than *Synechocystis* GlgA1 and GlgA2, consistent with previous reports [22]. These results confirm the assay's reliability across different GlgA enzymes, while highlighting the substantially slower catalytic rates of *Synechocystis* isoenzymes relative to *E. coli*.

### Primer architecture dictates GlgA activity profiles

Next, we assessed the impact of primer structure on glycogen synthesis by testing maltose and maltotetraose as alternative short and unbranched primers, alongside comparisons with soluble potato starch (hereafter starch) and bovine glycogen to evaluate the effect of primer branching. Both GlgA1 and GlgA2 utilized the shorter, less‐branched primers, albeit with reduced activity compared to glycogen (Fig. 2C). No GlgA activity was observed in the absence of a primer. These results indicate that α‐1,4‐glycosidic bond formation by GlgA at non‐reducing ends strictly requires priming, with a preference for longer, branched glycan primers.

To further evaluate primer‐dependent modulation, starch and glycogen concentrations were titrated at near saturating ADP‐Glc levels (1 mm) (Fig. 2D). Both isoenzymes exhibited a rapid increase in activity with rising glycogen concentrations (EC_50_: GlgA1 = 26 ± 3.1 μg·mL^−1^; GlgA2 = 21 ± 2.2 μg·mL^−1^), whereas responses to starch were significantly weaker (EC_50_: GlgA1 = 236 ± 21 μg·mL^−1^; GlgA2 = 270 ± 37 μg·mL^−1^). Comparing maximal activities (*V*_max_), reactions using starch as a primer resulted in a reduction in GlgA1 activity by 16% and GlgA2 activity by 34% compared to glycogen. These data suggest that, despite comparable catalytic efficiencies, the isoenzymes differ in their primer interactions, potentially reflecting variant preferences for primer structure or branching.

### GlgB‐driven branching preferentially enhances GlgA2 activity

**Fig. 3:**
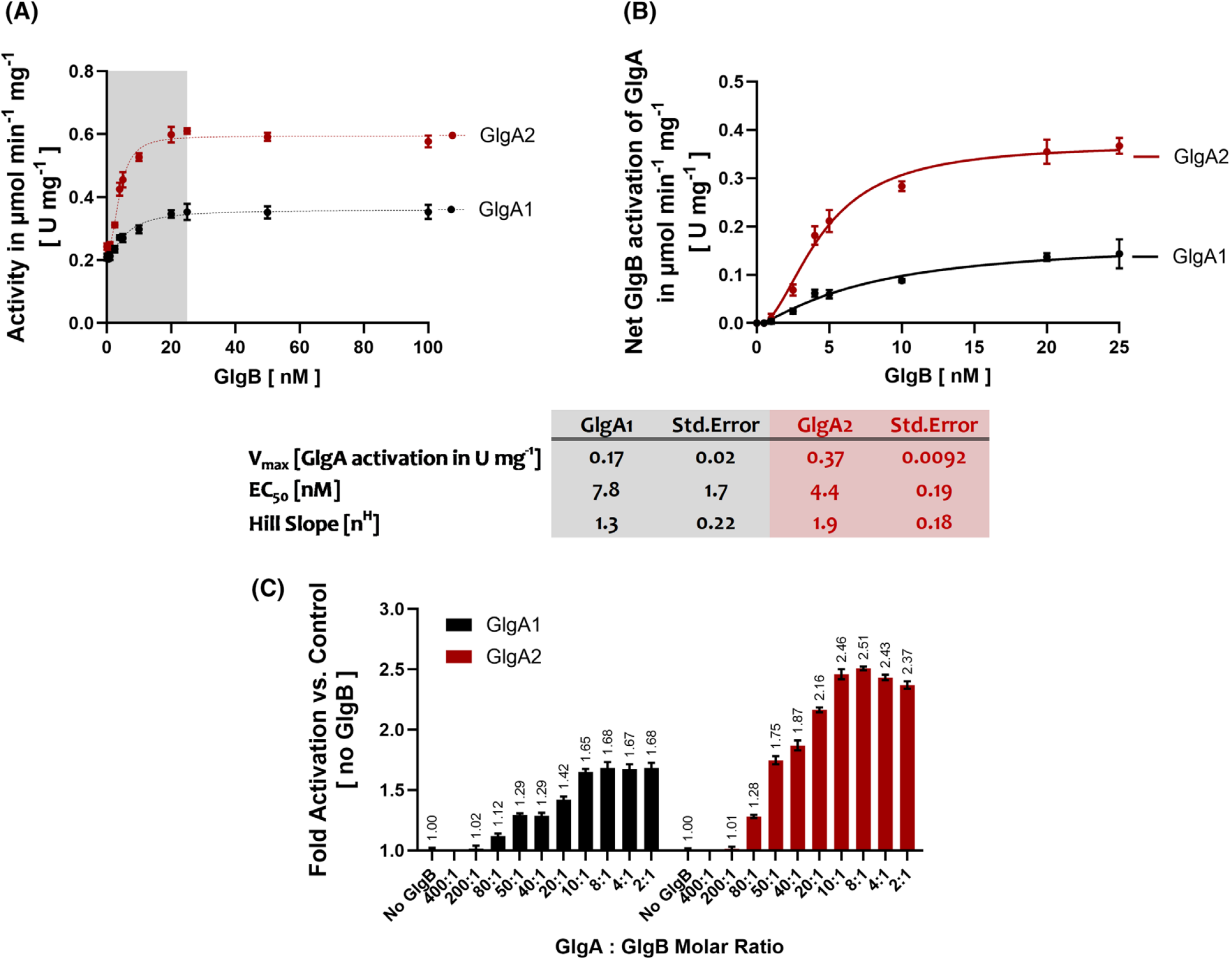
Activation of GlgA isoenzymes by GlgB. (A) GlgA enzymatic activity with GlgB. The assay was performed using increasing concentrations of GlgB with 200 μg·mL^−1^ soluble starch as the synthesis primer. Reactions were initiated by adding 1 mm ADP‐Glc. The area shaded in gray is represented in panel (B). (B) Net activation of both GlgA isoenzymes (200 nm GlgA) was assessed with increasing GlgB concentrations in the presence of 200 μg·mL^−1^ soluble starch as the synthesis primer. Reactions were initiated by adding 1 mm ADP‐Glc. Net activity values on the *y*‐axis represent GlgA activity after subtracting the baseline activity measured without GlgB from each corresponding data point on the *x*‐axis. (C) The fold change in GlgA activity was calculated relative to a control reaction without GlgB. GlgB concentrations are expressed as molar ratios to 200 nm GlgA isoenzyme, with the optimal GlgA: GlgB ratio determined to be 10 : 1. Raw activity values are presented in panel (A). Where applicable, each data point represents the mean of replicates with the SD shown as error bars (*n* = 3–6).

Next, we introduced the branching enzyme GlgB into the glycogen synthesis reaction to promote primer branching and examined its effects on GlgA activity. Both GlgA isoenzymes exhibited noticeable activation with increasing GlgB concentrations up to around 20 nm GlgB or a molar ratio of one GlgB to ten GlgA monomers (Fig. 3A). Fig. 3B illustrates the net activation (activity normalized against a control without GlgB) of both GlgA isoenzymes with increasing concentrations of GlgB, using starch as a representative of an unbranched primer, demonstrating at least a twofold enhancement in their activity. However, the maximum activation of GlgA1 (increase of 0.17 ± 0.02 U·mg^−1^) was substantially lower than that of GlgA2 (increase of 0.37 ± 0.01 U·mg^−1^). Furthermore, GlgA1 required almost twice the amount of GlgB for half‐maximal activation (EC_50_: 7.8 ± 1.7 μm) compared to GlgA2 (EC_50_: 4.4 ± 0.19 μm). These results suggest that GlgB activation of GlgA2 is slightly more efficient than that of GlgA1, which is further supported by the Hill coefficient for GlgA2 (*n* ^H^: 1.9 ± 0.18) compared to GlgA1 (*n* ^H^: 1.3 ± 0.22), indicating positive cooperativity between GlgB and GlgA2 (Fig. 3C).

**Fig. 4:**
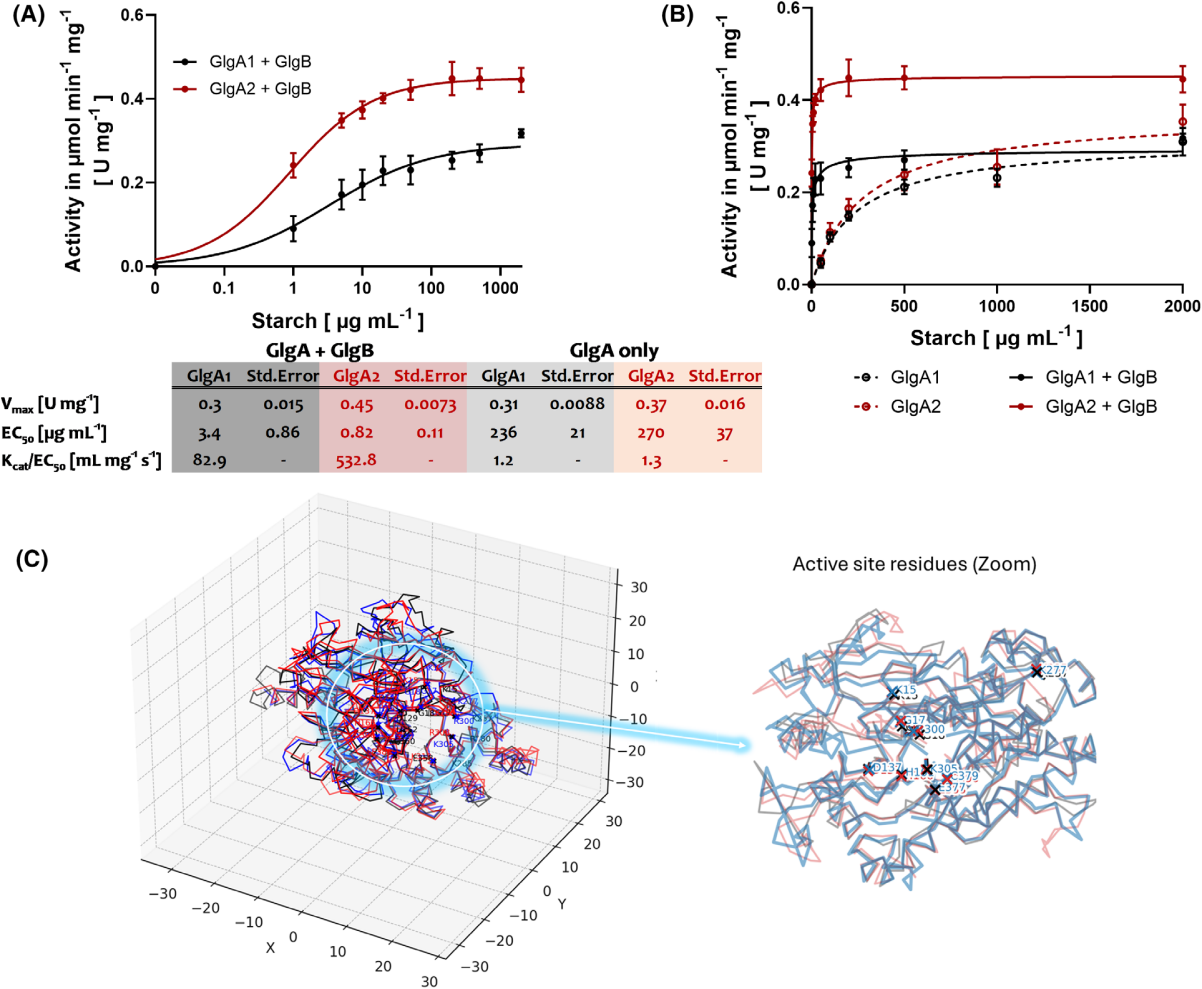
Effect of branching on GlgA primer sensitivity and structure‐guided motif mapping of GlgA. (A) Starch titration in the presence of 20 nm GlgB and 200 nm GlgA (1 : 10 molar ratio of GlgB to GlgA) with 1 mm ADP‐Glc, showing the effect of primer concentration on GlgA activity. (B) Overlay of starch titration curves with GlgB (solid lines) and corresponding starch titration data from Fig. 2C (dashed lines) for direct comparison. (C) Structure‐based superposition of GlgA1 and GlgA2 onto GlgA^*E.coli*^ shown as Cα traces (GlgA^*E.coli*^, blue; GlgA1, gray; GlgA2, red). Positions of key catalytic/substrate‐binding residues (K15, G17, G18, D137, H161, K277, R300, K305, E377, C379; GlgA^*E.coli*^ numbering) are indicated by crosses at the corresponding Cα atoms, with residue labels color‐coded by protein (blue/gray/red). The active site pocket is highlighted (circle) and shown as a magnified view on the right to emphasize local structural conservation. Where applicable, each data point represents the mean of replicates with the SD shown as error bars (*n* = 3–12).

To elucidate the modulatory effects of GlgB on GlgA isoenzyme activity, we tested increasing concentrations of starch in the presence of both enzymes to identify the primer concentration at which GlgB‐mediated branching most effectively potentiates GlgA catalytic performance. In the presence of GlgB, the EC_50_ for starch was approximately fourfold lower for GlgA2 than GlgA1, accompanied by a higher *V*_max_ for GlgA2 (Fig. 4A). More strikingly, when GlgB cooperates with either GlgA isoenzyme, the EC_50_ for starch decreased by at least 70‐fold for GlgA1 and 330‐fold for GlgA2 compared to reactions without GlgB (Fig. 4B). Interestingly, the maximum activity of GlgA1 at saturating starch concentrations remained essentially unchanged by GlgB (*V*_max_ with GlgB: 0.30 ± 0.02 U·mg^−1^ vs. without GlgB: 0.31 ± 0.01 U·mg^−1^). In contrast, GlgA2 exhibited a 21.6% increase in maximum activity in the presence of GlgB (*V*_max_ with GlgB: 0.45 ± 0.01 U·mg^−1^ vs. without GlgB: 0.37 ± 0.02 U·mg^−1^). Moreover, the catalytic efficiencies (defined as *K*_cat_/Primer EC_50_) of both enzymes increased by at least two orders of magnitude in the presence of GlgB, highlighting the substantial impact of branching on enzyme performance (Fig. 4). Notably, the catalytic efficiency of GlgA2 with GlgB was 6.4‐fold higher than that of GlgA1, underscoring a strong dependence of GlgA2 activity on cooperative interactions with GlgB. Taken together, these findings indicate that GlgB significantly enhances the catalytic efficiency and substrate affinity of both GlgA isoenzymes, with a particularly pronounced effect on GlgA2, while GlgA1 activity at higher primer concentrations appears to be more influenced by the overall primer concentration rather than maximum activation by GlgB alone.

### Comparative capacities identify GlgA as the rate‐limiting step in *Synechocystis*

To evaluate how coupling to the comparatively slower *Synechocystis* GlgA isoenzymes affects GlgC (as compared to GlgA^*E.coli*^), we compiled previously reported kinetics parameters for GlgC measured by malachite green (MG) Pi‐release assay together with those for GlgA reported above. We compared the intrinsic catalytic capacities on a common flux scale (μm·min^−1^) to relate their activities directly. Using the MG assay‐derived specific activity of GlgC (8.9 U·mg^−1^ at 4.98 nm GlgC tetramer) as a benchmark for supply, and the Assay A specific activities (*V*_max_) of GlgA1, GlgA2 and GlgA^*E. coli*^ as measures of utilization capacity, we estimated the minimum GlgC concentration required to match GlgA demand under assay conditions (Table 2) [6]. For *Synechocystis*, our calculations indicate that matching the demand of 200 nm GlgA would require on the order of ~3.2 nm GlgC for GlgA1 (≈62 GlgA1 monomers per GlgC tetramer) or ~5 nm GlgC for GlgA2 (≈40 GlgA2 monomers per GlgC tetramer), whereas the highly active GlgA^*E. coli*^ would require over 3.33 μm GlgC tetramer to match 200 nm of GlgA^*E.coli*^ (≈0.06 : 1). Thus, *Synechocystis* GlgA remains the kinetic bottleneck over a wide range of realistic GlgA:GlgC ratios, while GlgA^*E. coli*^ would be strongly supply‐limited by GlgC.

### Structural alignment reveals conserved active site geometry despite pronounced global backbone divergence in *Synechocystis* GlgA

To further investigate this difference in utilization capacity between *Synechocystis* and *E. coli* GlgA, we performed a structure‐guided motif mapping using AlphaFold models (Fig. 4C). Cα‐RMSD‐based structural alignment revealed closer overall structural correspondence between GlgA^*E. coli*^ and GlgA2 than between GlgA^*E. coli*^ and GlgA1 (3.19 Å/469 residues vs. 6.06 Å/448 residues), consistent with larger backbone deviations and/or domain‐level differences in GlgA1 (Table 3). Despite substantial global backbone differences in both *Synechocystis* GlgA, we managed to identify previously reported conserved residues linked to essential roles in catalysis and substrate positioning in GlgA^*E. coli*^ (Table 4) [23, 24, 25, 26]. The active site region was found to be highly conserved: after fitting on residues within 6 Å of key catalytic/binding residues, the active site Cα RMSD was 0.68 Å (GlgA1) and 0.94 Å (GlgA2), and the 10 key residues alone superposed with ≤ 0.35 Å RMSD (Table 3). Within the tight active site pocket, ≤ 6 Å from the 10 key residues, backbone superposition showed that GlgA^*E. coli*^ and GlgA1 are nearly indistinguishable (mean backbone RMSD 0.47 Å; 93.9% of residues ≤ 1.0 Å), with only minor, largely conservative local shifts (≤ 1.32 Å) that are unlikely to account for large kinetic differences (Table 5). In contrast, GlgA^*E. coli*^ vs. GlgA2 displayed a generally conserved pocket (mean 0.65 Å; 83.8% ≤ 1.0 Å) but contained two localized perturbation hotspots: a pronounced deviation centered on the A329–G330 region (A329 → A338 and G330 → T339; backbone RMSD ~3.5 Å) and a second cluster around Q281–I282 (Q281 → R289 and I282 → E290; backbone RMSD ~1.7–2.1 Å) (Table 5). These deviations combine substantial backbone displacement with non‐conservative side‐chain changes, consistent with altered loop flexibility and electrostatics at the pocket periphery that could hypothetically modulate active site closure and/or acceptor/donor positioning, all the while the catalytic core itself remains structurally conserved.

**Table 3:** GlgA active site Cα‐RMSD.

	Region	Residues [*n*]	Cα‐RMSD [Å]
GlgA^*E.coli*^ vs. GlgA1	Global	448	6.06
	Key residues only (10)	10	0.35
	Active site pocket (6 Å from key residues)	99	0.68
GlgA ^*E.coli*^ vs. GlgA2	Global	469	3.19
	Key residues only (10)	10	0.25
	Active‐site pocket (6 Å from key residues)	99	0.94

**Table 4:** GlgA key active site residue position mapping.

*E. coli* Residue	GlgA1 equivalent	GlgA2 equivalent	Reported Function in GlgA^*E. coli*^
K15	K15	K15	Conserved KXGG loop [25]
G17	G17	G17	Conserved KXGG loop [25]
G18	G18	G18	Conserved KXGG loop [25]
D137	D129	D137	Acceptor (glucan chain) positioning [26]
H161	H152	H166	Major catalytic‐geometry/transition‐state contributor [26]
K277	K257	K285	Catalytic contribution [24]
R300	R280	R308	Catalytic phosphate‐handling network [26]
K305	K285	K313	Catalytic phosphate‐handling network [26]
E377	E358	E388	Glucose positioning [23]
C379	C360	C390	Donor sugar/phosphoglucose recognition [23]

**Table 5:** Per‐residue backbone RMSD (Å) over N, Cα, C, O after a local least‐squares fit using the 99 pocket Cα atoms.

Largest local deviations (top 5)				
GlgA^*E.coli*^ → GlgA1		Backbone RMSD (Å)	Cα deviation (Å)	GlgA^*E.coli*^ vs. GlgA1 (*n* = 99)
F197	V179	1.32	1.31	Mean 0.47 Å, median 0.40 Å
V6	V6	1.15	1.3	90th percentile 0.83 Å, 95th percentile 1.01 Å
D308	D288	1.08	1.07	Max 1.32 Å
V310	V290	1.05	0.92	93.9% of residues are ≤ 1.0 Å
L198	M180	1.01	0.9	

GlgA^*E.coli*^ → GlgA2		Backbone RMSD (Å)	Cα deviation (Å)	GlgA^*E.coli*^ vs. GlgA2 (*n* = 99)
A329	A338	3.56	3.33	Mean 0.65 Å, median 0.45 Å
G330	T339	3.54	3.4	90th percentile 1.23 Å, 95th percentile 1.50 Å
V310	V318	2.13	2.2	Max 3.56 Å
I282	E290	2.05	1.97	83.8% of residues are ≤ 1.0 Å
Q281	R289	1.71	1.61	

### GlgA1 produces more‐branched glycogen, whereas GlgA2 yields a more linear polymer

To study the products of the glycogen synthesis reaction in detail, we repurposed the Assay A system for preparative purposes by omitting the coupling system components for the spectrophotometric assays, namely LDH and NADH (Fig. 1). To complete the *in vitro* glycogen synthesis pathway, we introduced an upstream ADP‐Glc production module by integrating GlgC at a stoichiometric ratio of 1 : 1 GlgA monomer to GlgC tetramer and started the glycogen synthesis reaction via addition of Glc‐1P, using starch as the synthesis primer (Assay B).

**Fig. 5:**
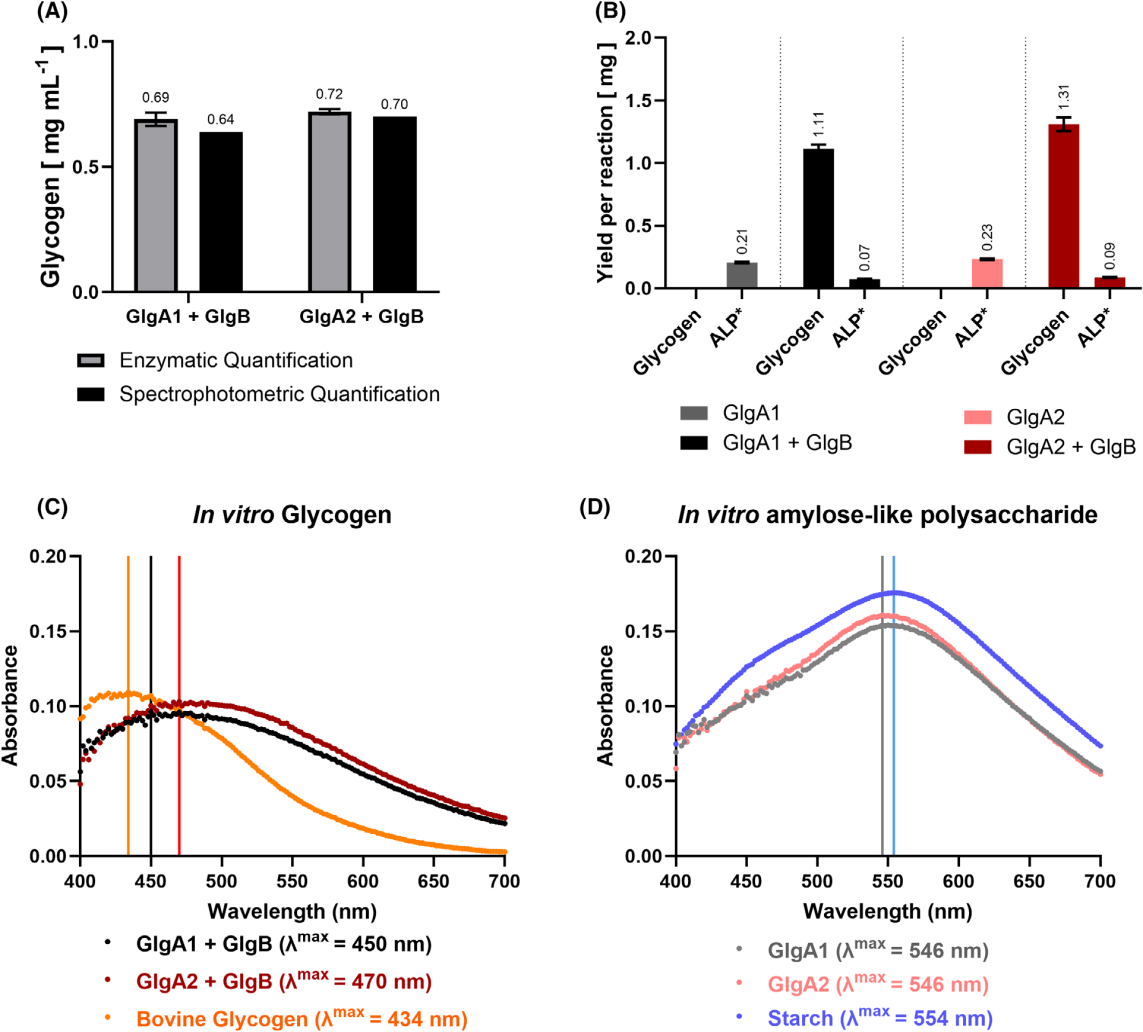
Characterization of *in vitro* polymer products (Assay B). (A) Comparison of glycogen quantification methods. Pooled glycogen samples from Assay B replicates (*n* = 6) were adjusted to 1 mL volume. Glycogen concentrations were first measured using the iodine‐iodide spectrophotometric assay and subsequently validated by an enzymatic rapid glycogen assay. Paired *t*‐test analysis yielded a *P* value of 0.2578, indicating no significant difference (*P* > 0.05). (B) Yields of reaction products from Assay B, expressed as mg of product per 1 mL reaction volume. Each data point represents the mean of six replicates (*n* = 6), with standard deviation (SD) shown as error bars. Absorbance spectra between 400 and 700 nm of pooled synthesis products from Assay B. Equal volumes of replicate reactions from (B) were combined, and concentrations were adjusted to 0.5 mg·mL^−1^ for glycogen samples and 0.1 mg·mL^−1^ for amylose‐like samples. Maximum absorbance values (*λ* ^max^) were calculated using the Area Under the Curve (AUC) function in GraphPad Prism, referencing the highest peak identified, and are indicated by vertical lines on the *x*‐axis. * Amylose‐like Polysaccharides (ALP). (C) Absorbance spectra of glycogen samples from Assay B compared to the bovine glycogen standard. (D) Absorbance spectra of amylose‐like samples from Assay B compared to a starch standard.

Products formed in the presence of GlgB were considered predominantly branched glycogen, whereas those produced in its absence were considered amylose‐like linear glucans. Polymer synthesis products from Assay B were quantified by both iodine‐iodide spectrophotometric staining and enzymatic determination (Fig. 5A), yielding consistent results with no significant differences observed between the two quantification methods. Figure 5B presents the synthesis product yields for both GlgA isoenzymes with and without GlgB. From 1 mL reaction mixtures, GlgA2 in combination with GlgB produced 18.1% more glycogen (1.3 mg) than the GlgA1‐GlgB reaction (1.1 mg). Similarly, in the absence of GlgB, GlgA2 synthesized 12.9% more amylose‐like polymer (0.233 mg) compared to GlgA1 (0.206 mg). Notably, reactions lacking GlgB demonstrated a substantial decrease in product yield, with an 82.9% reduction for GlgA1 and 81.3% for GlgA2.

**Fig. 6:**
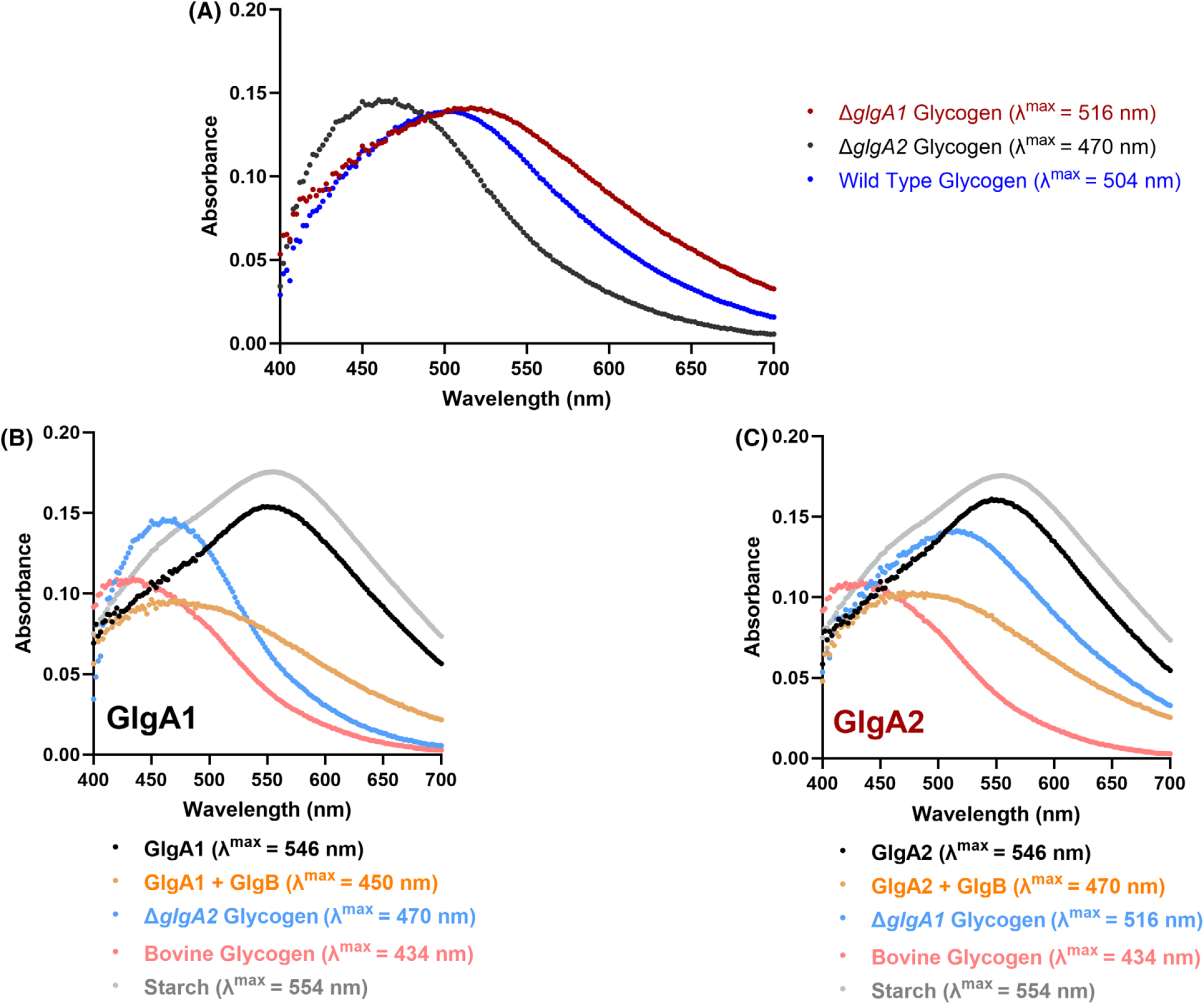
Comparison of *in vitro* polymer products with *in vivo* glycogen. Absorbance spectra between 400 and 700 nm of pooled synthesis products from Assay B were compared to glycogen extracted from *Synechocystis* wild‐type and *glgA*‐deficient mutant cultures. Equal volumes of replicate reactions from Assay B or isolated glycogen from *Synechocystis* cultures were combined, and concentrations were adjusted to 0.5 mg·mL^−1^ for glycogen samples and 0.1 mg·mL^−1^ for amylose‐like samples. Maximum absorbance values (*λ* ^max^) were calculated as previously described using the Area Under the Curve (AUC) function in GraphPad Prism. (A) Absorbance spectra of glycogen isolated from Δ*glgA2*, Δ*glgA1*, and wild‐type *Synechocystis* cultures. (B) Comparison of GlgA1‐derived synthesis products with bovine glycogen, starch, and glycogen isolated from GlgA2‐deficient (Δ*glgA2*) *Synechocystis* mutants. (C) Comparison of GlgA2‐derived synthesis products with starch, bovine glycogen, and glycogen isolated from GlgA1‐deficient (Δ*glgA1*) *Synechocystis* mutants.

Analysis of iodine‐iodide absorbance spectra revealed subtle differences in polymer branching between products of the two GlgA isoenzymes in the presence of GlgB (Fig. 5C). Glycogen synthesized by GlgA1 showed an absorbance maximum (λ^max^) of 450 nm with higher absorbance at lower wavelengths, indicative of a more‐branched structure, approximating the profile of highly branched bovine glycogen (*λ* ^max^: 434 nm). Conversely, GlgA2‐derived glycogen exhibited a *λ* ^max^ of 470 nm, suggestive of comparatively fewer branches. The amylose‐like polysaccharides generated in the absence of GlgB from both isoenzymes showed *λ* ^max^ values near 546 nm (Fig. 5D), characteristic of linear starch‐like molecules, closely matching the profile of the starch standard *λ* ^max^ of 554 nm. We further compared these *in vitro* polymer profiles with glycogen isolated from *Synechocystis* mutants deficient in either GlgA1 (Δ*glgA1*) or GlgA2 (Δ*glgA2*) (Fig. 6A). Glycogen from the Δ*glgA2* mutant displayed a *λ* ^max^ of 470 nm, resembling bovine glycogen (Fig. 6B), whereas glycogen from Δ*glgA1* had a higher *λ* ^max^ of 516 nm, aligning more closely with starch (Fig. 6C).

Although neither mutant‐derived glycogen spectrum perfectly matched their respective *in vitro* synthesized products, the glycogen produced *in vivo* by GlgA1 exhibited spectral properties (*λ* ^max^ = 470 nm) closely resembling those of *in vitro* GlgA1‐derived glycogen (*λ* ^max^ = 450 nm). Both forms demonstrated a higher degree of branching compared to glycogen produced by GlgA2. *In vivo* synthesized glycogen by GlgA2 displayed starch‐like characteristics with a notably higher *λ* ^max^ of 516 nm, indicating less branching. This trend of reduced branching was also observed in glycogen synthesized *in vitro* by GlgA2, though less pronounced, as reflected by a *λ* ^max^ of 470 nm. Wild‐type glycogen showed intermediate properties, with a *λ* ^max^ of 504 nm, positioning it between the two mutant profiles and recapitulating the distinct structural divergences between GlgA1‐ and GlgA2‐derived glycogen *in vivo*. (Fig. 6A).

### Modified assay a enables quantification of PGM forward reaction kinetics

To further expand the utility of our assay system, we modified the core assay (Assay A) to examine PGM activity by coupling the PGM reaction to equimolar GlgC and GlgA^*E.coli*^ (Assay C) (Fig. 1). PGM catalyzes the reversible interconversion of Glc‐1P and Glc‐6P. *Synechocystis* bears two genes encoding putative PGM enzymes (*sll0726* and *slr1334*); the *sll0726*‐encoded enzyme was selected for this study, as it represents the primary PGM isoform in *Synechocystis* [16]. To ensure substrate turnover was not rate‐limited, the coupling enzymes GlgC and GlgA^*E.coli*^ were included in the reaction at 400 nm each, placing the GlgA:GlgC ratio at 1 : 1, far above the ideal 0.06 : 1 established previously (Table 2).

**Fig. 7:**
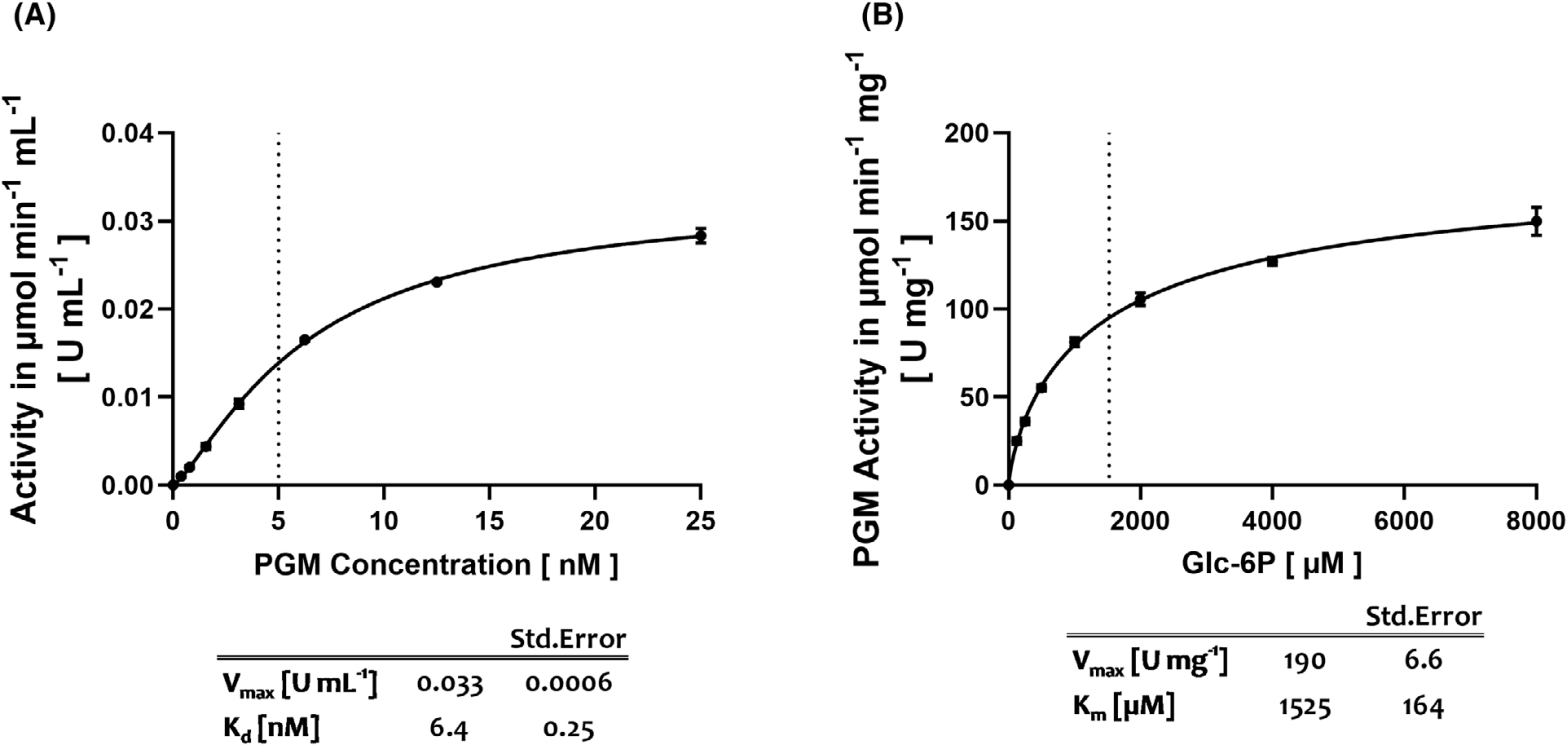
PGM forward reaction Assays (Assay C). (A) Titration of PGM at the indicated concentrations. Reactions were initiated by the addition of 1 mm Glc‐6P. Activity values (U·mL^−1^) on the y‐axis represent the overall enzymatic activity of the coupled pathway, including Glc‐1P formation from Glc‐6P, subsequent ADP‐Glc synthesis, and glycogen polymerization. The dotted line on the *x*‐axis denotes the standard PGM concentration used in subsequent assays. (B) Titration of Glc‐6P in the presence of 5 nm PGM. The *K*_m_ value is indicated by the dotted line on the *x*‐axis. Where applicable, each data point represents the mean of independent replicates with the SD shown as error bars (*n* = 3–6).

A titration of PGM was performed to identify the concentration range in which the measured activity reflected intrinsic enzyme catalysis (Fig. 7A). The reaction rate increased linearly with PGM concentration up to the tested concentration of 6.13 nm, indicating that within this range, the observed activity corresponded directly to PGM turnover. Together with the determined dissociation constant (*K*_d_: 6.4 ± 0.25 nm), these results indicate that enzyme concentrations within this range are optimal for reliable kinetic measurements under the applied conditions. Subsequently, a Glc‐6P titration was conducted using 5 nm PGM. As shown in Fig. 7B, the resulting kinetic parameters demonstrated that the assay effectively resolved PGM catalytic properties, yielding a *V*_max_ of 190 ± 6.6 U·mg^−1^ and a *K*_m_ of 1.52 ± 0.16 mm for Glc‐6P.

## Discussion

We established an *in vitro* model of glycogen synthesis pathway from *Synechocystis* (Fig. 1). This model elucidates critical factors controlling glycogen synthesis, including primer selectivity and the modulatory role of GlgB. Additionally, we demonstrated production of glycogen *in vitro* and characterized structural differences between products synthesized by GlgA1 and GlgA2.

### Primer architecture and GlgB cooperation partition elongation labor between GlgA isoenzymes

The kinetic analyses demonstrate that GlgA1 and GlgA2 isoenzymes possess broadly similar catalytic efficiencies despite subtle differences in their kinetic parameters, implying differential optimization potentially aligned with distinct cellular or metabolic contexts. Both isoenzymes can utilize short, linear polysaccharide primers, though less efficiently than branched glycogen, indicating some flexibility in primer selectivity but also emphasizing that primer structure notably influences enzymatic activity. The absence of detectable GlgA activity without an exogenous primer (Fig. 2C) demonstrates that *Synechocystis* GlgA are strictly primer‐dependent and therefore unlikely to function as initiating enzymes. Together with the reported requirement for an unidentified protein factor for *de novo* glycogen initiation *in vivo*, our results suggest that initiation may involve an upstream priming/acceptor‐generating activity that supplies the first α‐glucan substrate for GlgA [27].

The distinct responses of GlgA1 and GlgA2 to starch and glycogen highlight differential primer interactions. Significantly higher concentrations of linear starch were needed to reach activities comparable to those with branched glycogen, indicating that branched primers with more readily accessible nonreducing ends are essential for optimal GlgA binding and activation (Fig. 2C,D). This highlights the role of GlgB in glycogen synthesis, where the inclusion of GlgB markedly enhanced the catalytic activities of both GlgA isoenzymes, albeit to different extents (Fig. 3). GlgA2, which synthesizes longer glucan chains, is suggested to be particularly susceptible to steric hindrance within the growing glycogen network [5, 10, 28]. The branching activity of GlgB alleviates these steric constraints by creating new branch points, thereby enabling GlgA2 to extend even longer chains more efficiently [10]. Consequently, GlgA2 exhibited a substantially stronger and more persistent activation in the presence of GlgB, indicative of a highly efficient functional cooperativity between the two enzymes (Fig. 3B). This pronounced synergy likely reflects a close coordination of elongation and branching reactions that supports continuous and effective glucan synthesis. In contrast, GlgA1 performs a more dispersive elongation, resulting in the synthesis of glycogen that is inherently more branched than that produced by GlgA2 [5, 10]. Accordingly, GlgA1 activity was observed to be increased only up to a defined level and then plateaued in the presence of GlgB, suggesting that the additional branching generated by GlgB does not further stimulate catalysis beyond a certain limit (Fig. 3). Additionally, at elevated GlgB concentrations, a slight decrease in GlgA2 activity was observed (Fig. 3C), an effect absent in GlgA1. This pattern suggests that GlgA2 may be particularly sensitive to steric effects arising from excessive enzyme crowding or suboptimal access to elongation sites despite branching's general role in relieving steric hindrances. Taken together, these observations highlight that GlgA2 engages in significantly more efficient and finely tuned cooperation with GlgB than GlgA1. The iodine‐iodide spectra of products formed with GlgB further reinforce that GlgA1 glycogen is more branched than GlgA2 glycogen despite similar primer usage [5, 27].

This aligns the observations from our enzyme assays with the physicochemical differences observed between glycogen synthesized by the two GlgA isoenzymes: glycogen produced by GlgA1, both *in vivo* and *in vitro*, is notably more branched than that formed by GlgA2 (Figs 5C and 6A). In the absence of GlgB, both isoenzymes generate amylose‐like, largely linear polymers with largely indistinguishable iodine–iodide spectral profiles, likely reflecting some degree of synthesis inhibition or a drastic reduction in GlgA activity to negligible levels over longer durations of sustained synthesis in the absence of branching (Fig. 5D). Taken together with the markedly lower yields of amylose‐like polysaccharides in reactions without GlgB, it is reasonable to conclude that branching is also essential to relieve steric hindrances that occur during sustained glycogen synthesis (Fig. 5B,D) [28]. These findings demonstrate that the role of GlgB extends beyond primer branching—it also plays a key role in glycogen structure reorganization for efficient GlgA activity and allows for efficient sustained glycogen synthesis.

The structural distinctions in glycogen produced by each GlgA isoenzyme, in addition to the characteristics of the isoenzymes themselves may reflect specialized physiological roles. For example, the short, highly branched glycogen synthesized by GlgA1 could confer survival advantages under environmental stresses, as observed in various bacteria where such glycogen is degraded more slowly, supporting reduced metabolic rates and prolonged viability, given that the shorter branches are more easily accessible to glycogen degradation enzymes [13]. In *Synechocystis*, the dependence of resuscitation from nitrogen starvation on GlgA1 supports the notion that GlgA1‐derived glycogen contributes to adaptability and survival, whereas GlgA2‐generated glycogen primarily could serve as a carbon storage molecule during conditions of high carbon flux [14, 27, 29]. Given that sustained glycogen synthesis occurs under such conditions, it is reasonable to assume that the reorganization of the growing glycogen granule by GlgB is especially crucial to support optimal GlgA2 activity. Since wild‐type glycogen exhibits characteristics intermediate between those of GlgA1‐ and GlgA2‐derived glycogen, it is reasonable to conclude that both isoenzymes cooperatively contribute to glycogen synthesis during normal growth, balancing metabolic flexibility with carbon storage needs (Fig. 6A) [10].

### Divergent glycogen synthesis control in phototrophs and heterotrophs

Oxygenic phototrophs—including *Synechocystis*—allocate primary control upstream at GlgC, which is activated by 3‐PGA and inhibited by Pi [6, 7, 8, 9], and diversify elongation via two GlgA isoenzymes with distinct operational biases [5, 27]. Additionally, given the roughly comparable *in vivo* expression levels of GlgA monomers and GlgC tetramers during normal heterotrophic growth, our observations also suggest an additional layer of intrinsic regulation of glycogen synthesis via enzyme stoichiometry [29, 30, 31]. Our *in vitro* reconstitutions support this proposed architecture in that: (i) GlgB sharply potentiates both GlgA isoenzymes under low‐primer conditions but disproportionately enhances GlgA2, (ii) product spectra indicate that GlgA1 yields more highly branched glycogen (lower *λ* ^max^) than GlgA2, and (iii) at a practical and biologically relevant 1 : 1 GlgC tetramer: GlgA monomer ratio, pathway flux is GlgA‐limited due to the lower intrinsic activity of the *Synechocystis* GlgA isoenzymes. In sum, primer/branching governance and modest GlgA catalysis—rather than maximal elongation throughput—determine the glycogen synthesis flux and polymer architecture in *Synechocystis*.

By contrast, in heterotrophic bacteria such as *E. coli*, glycogen synthesis is configured for rapid accumulation. GlgA^*E.coli*^ has high catalytic capacity *in vitro* [22, 32], and the GlgA–GlgB pair sustains appreciable elongation even when exogenous primer is scarce—under ‘unprimed’ conditions, GlgB (further facilitated by citrate) supports ~30% of the GlgA^*E.coli*^ *V*_max_ otherwise achieved with saturating glycogen primer concentrations [33, 34]. *In vivo*, the maltose/maltodextrin network (MalQ/MalP and associated transporters) continually supplies maltooligosaccharide acceptors, minimizing primer limitation and amplifying the impact of a fast GlgA [35, 36]. These features indicate an evolutionary reason why *E. coli* GlgC (GlgC^*E.coli*^) exhibits substantially higher intrinsic activity than the *Synechocystis* isoenzymes, and why GlgA^*E.coli*^ activity exceeds GlgC^*E.coli*^ by several fold [6, 32, 37].

Multiple sequence alignment and structure‐guided motif mapping indicate that both *Synechocystis* GlgA enzymes retain the canonical GlgA active site architecture. Residues classically implicated in ADP‐glucose binding and catalysis in GlgA^*E.coli*^—including the N‐terminal KXGG signature and other conserved catalytic/structural positions—are preserved at alignment‐equivalent sites (Table 4, Fig. 4C) [23, 24, 25, 26]. Consistent with this, pocket‐restricted superposition (≤ 6 Å from the ten key residues) shows that the immediate catalytic environment is highly conserved, most strikingly for GlgA1, whose tight‐pocket backbone differs from GlgA^*E.coli*^ only marginally (Table 5). Thus, the markedly lower apparent catalytic efficiency of *Synechocystis* GlgA relative to GlgA^*E.coli*^ is unlikely to reflect altered donor‐binding chemistry or loss of catalytic determinants within the pocket itself. Instead, our structural data support a model in which the difference in activity between GlgA^*E.coli*^ and *Synechocystis* GlgA is due to features of the global GlgA structure that govern how often the enzyme reaches (and maintains) a productive catalytic configuration. The larger whole‐structure deviations seen for GlgA1 (GlgA1 vs. GlgA^*E.coli*^), compared with GlgA2 vs. GlgA^*E.coli*^, suggest changes in interdomain orientation and/or hinge dynamics. These changes could slow formation of the closed, catalytically competent state and thus reduce overall catalytic activity (Table 3). GlgA2, while globally closer to GlgA^*E.coli*^, exhibits two localized perturbation hotspots at the pocket periphery that could further tune loop flexibility and electrostatics at the acceptor‐facing boundary, thereby modulating primer positioning, processivity, and substrate‐gated conformational transitions (Tables 3 and 5). Collectively, these observations argue that divergence in primer binding and conformational dynamics, rather than changes to the conserved catalytic core, provides the most likely explanation for the substantially lower activity of *Synechocystis* GlgA.

Supporting this is a further distinction in synthesis initiation and primer economy. Bacteria do not employ a glycogenin‐type protein primer; in some species, glycogen synthesis can initiate *de novo*, and primer pools arise via enzyme‐mediated interconversion of maltooligosaccharides [38]. This helps explain why *E. coli* can partly circumvent primer scarcity through GlgB‐assisted ‘unprimed’ synthesis and MalQ/MalP‐driven primer provision [34, 35, 36]. On the other hand, *Synechocystis* lacking a dedicated maltose circuit, relies more on primer economy and GlgB cooperation to maintain throughput. A similar control logic of GlgA‐GlgB coordinated elongation–branching is also documented in higher plants, where multiple starch synthase and branching enzyme isoforms assemble into catalytically competent complexes [39, 40]. Additionally, similar GlgA‐GlgC catalytic arrangements—higher intrinsic GlgC activity relative to starch/glycogen synthases—were also observed in various higher plants, hinting that these regulatory strategies might be common to photoautotrophs [1, 41, 42, 43, 44, 45]. Viewed together, these considerations provide a plausible mechanistic rationale: Despite largely working off the same molecular principles, *Synechocystis* GlgA operates in a measured kinetic regime that facilitates efficient GlgB‐mediated branch insertion under primer‐limited conditions, rather than being tuned for maximal elongation velocity *per se*, contrasting with the primer‐rich, high‐throughput configuration of *E. coli*.

### The coupled assay (Assay B) applications go beyond modeling glycogen synthesis

The coupled assay system developed in this study demonstrates considerable flexibility and can be readily adapted to address a broad range of biochemical questions beyond glycogen synthesis itself. Its modular design allows precise modification of reaction components to target different catalytic processes or synthetic outcomes. For example, scaling the assay for preparative purposes (Assay B, Fig. 5) highlights its utility for large‐scale *in vitro* glycogen synthesis, providing new perspectives on glucan structure formation and enzyme cooperation. Beyond analytical use, such adaptations may also serve practical applications, such as generating defined *Synechocystis* glycogen for downstream enzymatic studies—most notably of glycogen phosphorylase—without requiring extensive biomass cultivation.

Apart from applications directly related to glycogen synthesis, the adaptation applied in Assay C (Fig. 7) establishes a powerful approach for investigating phosphoglucomutase (PGM) activity and its regulation. While the kinetics of the reverse PGM reaction (Glc‐1P → Glc‐6P) have been thoroughly examined, the forward conversion (Glc‐6P → Glc‐1P) has received far less attention [16, 46]. By coupling GlgC and GlgA^*E.coli*^, our system enables reliable quantification of this underexplored reaction and could be easily expanded to screen for potential metabolic regulators influencing PGM activity.

Looking ahead, the modularity of the coupled platform provides a direct route to test flux control and compatibility hypotheses by systematically swapping pathway modules *in vitro* (e.g., replacing *Synechocystis* GlgA with a high‐turnover synthase such as GlgA^*E.coli*^) and quantifying how control shifts toward ADP‐glucose supply (GlgC) and energy status, as well as how elongation kinetics reshape glucan architecture through competition with branching/debranching. In parallel, an equally informative *in vivo* approach would be to complement a completely GlgA‐deficient *Synechocystis* background strain with GlgA^*E.coli*^ to test whether a fast elongation module is compatible with cyanobacterial glycogen homeostasis and stress physiology. Such a test is particularly interesting because complete loss of glycogen synthase activity in *Synechocystis* is reported to be lethal due to ADP‐glucose accumulation, with viability achievable only under specific conditions that redirect carbon flux [47]. Collectively, these applications underscore the assay's versatility as a platform for probing diverse aspects of carbohydrate metabolism and enzymatic regulation in a controlled *in vitro* context, as well as method to complement and inform additional physiological investigations.

## Conclusion

Our reconstruction of the *Synechocystis* glycogen biosynthesis pathway *in vitro* resolves glycogen synthesis into a hierarchical control scheme and a context‐dependent division of labor between GlgA isoenzymes. Upstream, GlgC governs precursor supply: Its substrate affinity and allosteric effectors tune ADP‐glucose availability and thereby gate entry into the pathway [6]. Downstream, GlgA imposes the principal flux limitation. Within this framework, primer architecture and GlgB cooperation partition elongation tasks between GlgA1 and GlgA2—branching density selectively accelerates GlgA2‐mediated chain extension, whereas GlgA1 favors shorter, more‐branched products—linking flux control to product fine structure. Finally, the coupled assay we established offers a modular platform to quantify control coefficients and to generalize these principles to other nucleotide‐sugar polymer pathways, such as resolving PGM forward kinetics.

## Author contributions

KL: designed and carried out the experiments and the wrote paper. DB: Assistance with Assay C. SD and KF: conceptualization, supervision, and paper writing. All authors contributed at varying stages in the editing and review of the paper.

## Data Availability

Enzymatic data and all figures presented in this study have been deposited here (https://doi.org/10.15490/fairdomhub.1.assay.2758.1) on the data and model management platform FAIRDOMHub [48].
